# The Validity of d′ Measures

**DOI:** 10.1371/journal.pone.0031595

**Published:** 2012-02-20

**Authors:** Astrid Vermeiren, Axel Cleeremans

**Affiliations:** Consciousness, Cognition and Computation Group, Université Libre de Bruxelles, Brussels, Belgium; University College London, United Kingdom

## Abstract

Subliminal perception occurs when prime stimuli that participants claim not to be aware of nevertheless influence subsequent processing of a target. This claim, however, critically depends on correct methods to assess prime awareness. Typically, d′ (“d prime”) tasks administered after a priming task are used to establish that people are unable to discriminate between different primes. Here, we show that such d′ tasks are influenced by the nature of the target, by attentional factors, and by the delay between stimulus presentation and response. Our results suggest that the standard d′ task is not a straightforward measure of prime visibility. We discuss the implications of our findings for subliminal perception research.

## Introduction

While the existence of unconscious perception is now endorsed by many authors (e.g. [1]), there is continuing debate about the extent of its influence (e.g., [2]–[Bibr pone.0031595-Lin1] [Bibr pone.0031595-Kanai1] [Bibr pone.0031595-VandenBussche1] [6]). Assessing awareness is obviously critical in making inferences about unconscious perception. Early subliminal perception research simply resorted to asking participants whether they could see the shortly presented stimuli or not. Such subjective methods, however, soon attracted considerable criticism [7], of which the most important was their lack of sensitivity: Participants are likely to fail to report conscious contents when they lack confidence in their perceptual judgements (“the underconfidence phenomenon”, e.g., [8]).

In this and other fields (e.g., implicit learning research, see [9]), researchers thus began preferring objective measures such as participants' ability to choose amongst several alternatives which stimulus they have been exposed to subliminally. Amongst such measures, the Signal Detection Theory sensitivity measure d′ [10] has now become the standard way of assessing awareness. A dissociation logic is applied: if primes exert an indirect influence on participants behavior (mostly shown by reaction times in an indirect task), but fail to reach awareness in a direct d′ test, one can argue that primes were unconsciously processed during the priming task.

Thus, after completion of the main priming task, participants are typically asked to perform a forced-choice recognition task on the prime stimuli. One can thus compute d′ for each participant, based on the z-scores for hit rates and false alarm rates. A d′ close to zero is interpreted as a lack of conscious access. Different d′ tasks have been used, such as detection tasks [11] or identification tasks [12]. After Holender's [13] critical review, d′ tasks have been further improved, specifically by ensuring that the priming task and the d′ task are as comparable as possible, as suggested by Reingold and Merikle [14]. Most modern paradigms aimed at exploring priming effects meet the first three criteria of Reingold and Merikle: Prime-target sequences are presented in exactly the same way in both phases (“task context” criterion), participants perform the same task twice, once on the targets and once on the primes (“stimulus states” criterion”), and should not be influenced by a response bias (“perceptual sensitivity” criterion”). However, Reingold and Merikle's fourth criterion (“same response metric”) is typically not fulfilled. Researchers often use a continuous metric for the priming task (e.g., reaction times) and a discrete measure for the awareness test (seen/not seen judgments).

There are reasons to believe, however, that the current focus on keeping the priming and the d′ tasks as comparable as possible has resulted in several potentially problematic issues insofar as assessing awareness is concerned. Thus, while we agree that the claim of unconscious perception is established, at least in a functional sense, when d′ is zero, one cannot claim that primes were actually not consciously perceived at the time they were presented. Other factors, such as target interference, might hinder participants' ability to report on primes they were weakly aware of at the time of presentation. We have identified three such factors.

The first concerns how attention is distributed. In a priming task, participants are told to ignore the primes and to focus on responding to the targets. In the d′ task, however, participants are told to focus on the primes while ignoring the targets. While this ensures that participants find themselves in the best conditions to identify the primes, and hence minimizes the likelihood of erroneously concluding that they were unaware of the primes, no study has assessed the influence of this factor on d′ performance. The influence of the distribution of attention has, however, been investigated for prime identification tasks. Thus, Dark [15] reported no difference in accuracy between a group that had to perform a target naming task and a prime identification on each trial compared to a group that had to perform only prime identification on each trial. In contrast, Dagenbach, Carr and Wilhelmsen [16] reported an influence of the type of measure of prime visibility (i.e., detection or discrimination versus semantic similarity) on the priming effect in a subsequent block, indicating that irrelevant attentional factors influence how primes are processed during the priming task. Here, we compare d′ performance for conditions in which participants are allowed to focus on the primes with conditions in which attention is divided between primes and targets.

Second, participants' responses in the d′ task are likely to be influenced by the presence of the target. This could increase the visibility of the primes when prime and target are semantically related [15], [17], [18]. However, the target could also result in decreased prime visibility because its processing interferes with processing of the prime. Crucially, impaired ability to inhibit a response to the target has little to do with prime awareness. Here, we approached this question by comparing performance in a standard d′ task with a d′ task in which targets were neutral and hence failed to elicit directed responses while nevertheless masking the primes in the same way.

Finally, participants' responses may depend on temporal factors, specifically the duration of the interval between prime presentation and response. If consciousness takes time [19], we would expect the weak traces resulting from prime presentation to grow stronger with increasing duration. We therefore manipulated the time (i.e., immediately vs. after an 800 ms delay) at which participants had to respond during the d′ task.

To summarize, we sought to systematically explore, in a single within-subjects design, the effects of attention, target valence, and temporal factors on prime visibility.

## Methods

### Ethics Statement

The study received ethics committee approval by the authorities responsible for our institution (ULB), Comité d'Ethique Facultaire Sciences Psychologiques et de l'Education. All participants have signed the informed consent.

### Participants

18 students (4 males, 14 females; mean age: 20 years) enrolled at the Université Libre de Bruxelles were compensated 7 euros for their participation.

### Material and stimuli

The experiment was carried out on a computer using E-prime version 2.0. Stimuli were displayed on a CRT monitor using a refresh rate of 75 Hz. Participants were seated 60 cm from the screen. Stimuli were adapted from Vorberg et al. [20]. Primes were black arrows pointing leftwards or rightwards shown on a white background. Primes subtended a visual angle of 1,72° width and 0,47° height. Targets were larger (subtending 3,44° width and 0,67° height) and also had a left- or rightward orientation. The mask was embedded in the target in the form of a white shape that covered the surface of both prime arrows. Neutral targets had the same appearance as arrow targets, but were rectangular and hence not directional.

### Procedure

**Figure 1:**
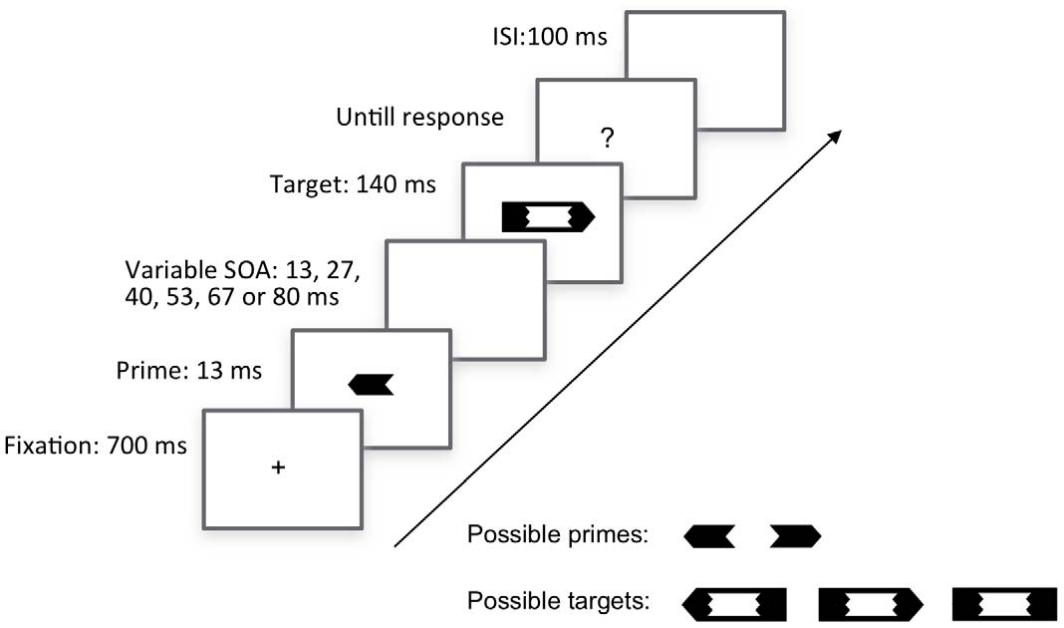
Trialprocedure.

The experiment started with a priming block in which people were kept unaware about the primes. Participants first performed 10 practice trials and were then asked to perform 6 blocks of 48 trials, with a short pause between each block. On each trial (Figure 1), a fixation cross was first presented for 700 ms. The prime arrow was then presented for 13 ms and was immediately followed by a blank screen of variable duration (13, 27, 40, 53, 67 or 80 ms). Immediately thereafter, the target arrow was presented for 140 ms. Both prime and target arrows were presented either 5% above or 5% below the middle of the screen. Finally, a question mark was displayed until participants had responded to the direction of the target arrow by pressing the most leftward or the most rightward key on the response box. Both reaction times and accuracy were recorded. The intertrial interval consisted of a blank screen displayed for 100 ms.

This design thus generates 48 trial types obtained by crossing SOA (6 levels), stimulus position (2 levels: above or below fixation), prime arrow direction (2 levels), and target arrow direction (2 levels). Each trial type was repeated 6 times and was presented in random order over the different blocks, resulting in a total of 288 trials per block.

After the priming task, participants were informed about the presence of the primes. An example of prime and target stimuli was shown and the experimenter made it clear that from now on the task would be to detect the orientation of the prime arrows. Each participant performed three awareness tests on three separate blocks. The first awareness test (“standard d′ test”) followed exactly the same design as the priming blocks. Only the instructions changed: Participants were now told to respond to the direction of the prime arrow instead of responding to the direction of the target arrow, which they were instructed to ignore.

In the second test (“neutral target test”), the same procedure was used but the target arrows were now replaced by the neutral target. In the third test (“switch test”), participants now had to identify the orientation of the prime arrows on half of the trials and the orientation of the target arrows on the other half of the trials. Which task had to be performed was indicated on a trial-to-trial basis through the identity of the target: People had to respond to the direction of the prime when the target was neutral, and to the direction of the target arrow when it was not.

The “standard d′ test” and the “neutral target test” each consisted of 144 trials (each trial type was repeated three times) presented in random order. The “switch test” consisted of 288 trials (144 prime identification trials and 144 target identification trials). The order of the tests was randomized across subjects. Finally, each test was administered twice, once with a delay and once without a delay. In the delay condition, the question mark was presented for 800 ms during which participants could not respond and were instructed not to do so. After 800 ms, the question mark became red as a signal for participants to respond. The order of the delay and non-delay block was counterbalanced across participants. Thus, a participant who had started one test in the delay condition started the other two tests with a delay. The entire experiment lasted 50 minutes.

## Results

### Priming

**Figure 2:**
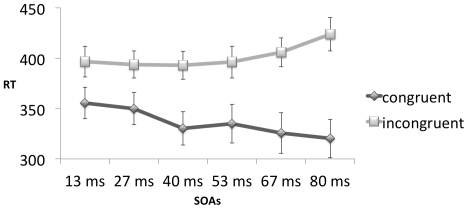
Mean RTs and SEs during priming blocks.

Figure 2 shows the median reaction times (RTs) elicited by congruent and incongruent stimuli presented at different SOAs during the priming task. RTs are slower for incongruent than for congruent trials, with the difference increasing with increasing SOAs.

These impressions were confirmed by an ANOVA with two within-subject factors: Congruency (2 levels: congruent vs. incongruent prime-target pairs) and SOA (6 levels: 13, 27, 40, 53, 67 or 80 ms). We observed a main effect of Congruency (F(1,17) = 85.52, p<.001, η^2^ = .83) with congruent trials eliciting faster responses (mean 336 ms, SE = 17 ms) than incongruent trials (mean 402 ms, SE = 14 ms) and a main effect of SOA (F(5,13) = 5.13, p<.01, η^2^ = .66). The interaction was also significant (F(5,13) = 7.27, p<.01, η^2^ = .74); the effect of congruency increased with increasing SOAs. Importantly, the congruency effect was significant for each SOA separately at a level of p<.001 as shown by separate contrasts.

### Awareness

**Figure 3:**
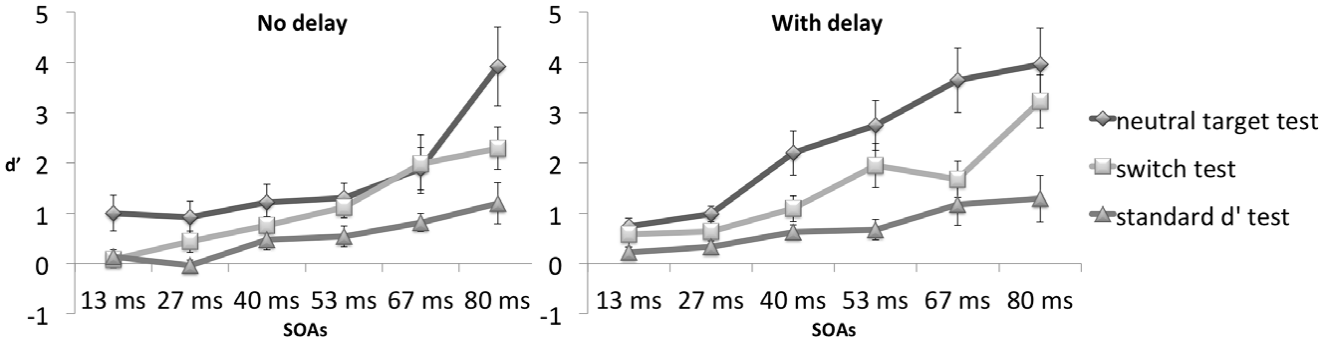
Mean d′s and SEs during awareness tests.

Next, we analyzed the results of the awareness tests. We first calculated d′ separately for each subject and each condition and averaged the computed values separately for each of the six conditions obtained by crossing the factors Type of Test (3 levels: Standard, Neutral Target, or Switch) and Delay (2 levels: 0 ms vs. 800 ms). The results appear in Figure 3, plotted separately for each SOA (6 levels: 13, 27, 40, 53, 67 or 80 ms). We analyzed the dataset by means of an ANOVA with three within-subject factors: Type of Test, Delay, and SOA. We observed a main effect of Delay (F(1,17) = 18.28, p = .001, η^2^ = .52, with larger d′s for the 800 ms condition (mean 1.54, SE = .21) than for the 0 ms condition (mean 1.11, SE = .20). There was also a main effect of SOA (F(5,13) = 6.76, p<.01, η^2^ = .72), with larger SOAs eliciting larger d′s. Finally, the main effect for Type of Test was also significant (F(2,16) = 17.23, p<.001, η^2^ = .68), with the highest d′ for the “neutral target test” (mean = 2.04, SE = .29), a lower d′ for the “switch test” (mean = 1.32, SE = .21) and the lowest d′ for the “standard d′ test” (mean = .62, SE = .16). We looked into more detail into the responses for the “standard d′ test”. They corresponded more often than chance level to the target response for the lowest SOA's (for SOA 13 ms: t(17) = 2.79, p = .01; for SOA 27 ms: t(17) = 2.81, p = .01; for SOA 40 ms: t(17) = 2.15, p<.05) and not for the highest SOA's. Two important contrasts were analyzed: d′ for the “neutral target test” was significantly higher than d′ for the “switch test” (F(1,17) = 17.82, p = .001, η^2^ = .51) and d′ for the “neutral target test” was significantly higher than d′ for the “standard d′ test” (F(1,17) = 34.80, p<.001, η^2^ = .67). The difference between the “neutral target test” and the “standard d′ test” was also significantly higher than the difference between the “neutral target test” and the “switch test” (F(1,17) = 26,92, p<.001, η^2^ = .61).

All two-way interactions (Delay*SOA (F(5,13) = 2.37, p = .10, η^2^ = .48, Delay*Type of Test test (F(2,16) = 1.31, p = .30, η^2^ = .14) and SOA*Type of Test (F(10,8) = 2.10, p = .15, η^2^ = .72) failed to reach significance. The three-way interaction Timing*SOA*Type of Test was, however, significant (F(10,8) = 3.56, p<.05, η^2^ = .82), with d′ values increasing more rapidly with SOA when there was a delay than when there was no delay, especially for the neutral target test.

To find out whether d′ was significantly different from zero for the lower SOAs, a t-test was performed for each condition separately. All values were significantly different from zero (threshold p<.05), except for the switch test at SOA 13 ms without a delay (t(17) = .44, p = .67), for the standard d′ test at SOA 13 ms without a delay (t(17) = .93, p = .37), for the switch test at SOA 27 ms without a delay (t(17) = 2.03, p = .06), for the standard d′ test at SOA 27 ms without a delay (t(17) = −.38, p = .71) and for the standard d′ test at 13 ms with a delay (t(17) = 1.87, p = .08).

## Discussion

We observed a clear influence of different variations of the d′ task on the resulting d′ values. First, dividing attention over prime and target decreased d′ values. This suggests that d′ values are overestimated when using the standard d′ task because participants are not required to pay attention to the primes during the priming blocks. They usually do not even know about their presence. Directing participants' attention to the primes leads to higher visibility of the primes. An alternative explanation could be that the divided attention block was easier, as suggested by higher overall accuracy (e.g., when considering prime and target identification together). Indeed, not only is it the case that the focus of attention differs between priming and target task, but the two tasks are also very different in how difficult they are to perform. This difference could for example demotivate participants. Congruently, Pratte & Rouder [21] observed an increase in performance accuracy in the awareness test when the test was made easier by mixing short prime presentations with longer presentations. Under such conditions, they even reported a lack of priming effects when controlling for difficulty, although this latter result was subsequently countered by Finkbeiner [22]. On the other hand, one could also argue that the divided attention block was more difficult, because task-switching was required. Whether task difficulty contributes to the explanation of our findings will have to be explored in further research.

However, a larger effect was observed for the presence of valenced targets during the d′ task. Participants are impaired in detecting the primes with valenced targets because they tend to report the direction of the targets instead of the direction of the primes. This results in an underestimation of d′ in the standard d′ task since failure to inhibit targets does not imply that the primes were not visible at the moment they were presented. Because this underestimation effect was larger than the overestimation effect, we can conclude that in general d′ values are underestimated using standard d′ tasks.

Further, we observed an effect of the timing of responses in the d′ task. Higher d′ values were observed when participants had to wait before responding. This response delay has been introduced by Vorberg et al. [20] and subsequently adopted by other research groups (e.g. [23]). The argument for using a delay is that responses in the d′ task are not only influenced by conscious processes, but also by unconscious information [24]–[Bibr pone.0031595-Kiesel1] [26]. Since unconscious processes generally exert their influence in a very short time window, the delay should diminish the influence of these unconscious processes and lead to a smaller d′ value. In contrast, we observed increased d′s with a delay. We hypothesize, congruently with Cleeremans & Sarrazin [27] (see also [19]), that this results from the fact that developing conscious representations takes time. Hence, increased — rather than decreased — accessibility is expected after a (short) delay.

Finally, it is important to note that our findings are based on metacontrast stimuli (adapted from [20]) rather than on the more popular pattern masking stimuli used in most priming research. Further experiments are necessary to establish whether our findings extend to such pattern masking methods. Further, we used an identification task as the awareness measure. However, other authors have argued that detection tasks are more sensitive [28]–[Bibr pone.0031595-Macmillan1] [30]. While we agree that detection is a more sensitive measure than identification *per se*, we argue that the identification task we have used is the correct measure in this context. As Snodgrass at al. [30] stated, the awareness measure “need not be sensitive to absolutely all conscious perception, but rather only to relevant conscious perception — namely, to the kind(s) of conscious perception that would be necessary at a minimum, for the effects of interest to occur” (p. 850). Here, the effect of interest is the influence of prime-target congruency on target reaction time. The minimum information necessary for such effects to occur is the identification of the direction to which the prime arrow is pointing. Merely detecting the presence of the arrow (arrow or no arrow?) may, in contrast, be based exclusively on low-level sensory details, such as luminance differences ([28]) and cannot result in congruency effects. Thus, a lack of awareness of prime direction (as established through identification) is sufficient to infer unconscious congruency effects.

### Conclusion

Recently, some have again argued for the subjective approach [31]–[Bibr pone.0031595-Dehaene3] [Bibr pone.0031595-Dienes1] [34]. However, this approach still faces the same issues as it did 50 years ago. Objective measures are essential when assessing awareness. We argue for a multi-pronged approach that involves both subjective and objective measures [35]. It is crucial, however, that the d′ task be correctly designed for inferences about unconscious processing to be valid. Our revised d′ task suggests the following: First, valenced targets should be avoided or replaced with neural targets. Second, attention on the primes should be distributed in the same way between the awareness test and the priming task. Finally, a delay should be provided so that representations of the weak prime stimulus can build up and their likelihood of becoming conscious representations is increased.
